# Borderline Ovarian Tumor during Pregnancy: A Case Report

**DOI:** 10.1155/2013/160319

**Published:** 2013-04-10

**Authors:** Joao Casanova, Raquel Maciel, Vânia Ferreira, Eugénia Fernandes, Rosa Maria Rodrigues

**Affiliations:** Department of Obstetrics and Gynecology, Porto Hospital Center, Porto 4050-371, Portugal

## Abstract

We report a case of a 33 year-old pregnant woman who was diagnosed at the time of the first trimester ultrasound with a multilocular solid arising form the right ovary. An abdominal MRI was performed afterwards and it revealed a pelvic mass, developing from the right ovary, with a liquid component but with a major solid area. CA 125 was within the normal range values. A laparotomy followed by right salpingo-oophorectomy was performed at 14 weeks of gestation and both the frozen section and the definitive histology revealed a borderline mucinous ovarian tumor. Ovarian tumors of low malignant potential comprise 10%–20% of all ovarian malignancies. They carry an excellent prognosis with 95%–99% long-term survival. Whereas in the past, radical surgery (hysterectomy and bilateral salpingo-oophorectomy with peritoneal staging) was standard regardless of the age of the patient, unilateral salpingo-oophorectomy with or without staging has become the recommended management for women who desire childbearing. In the absence of large prospective randomized trials it is difficult to know which are the best management practices and especially to determine the right moment during pregnancy to perform surgery in these patients.

## 1. Introduction

The incidental finding of an adnexal mass in pregnancy is becoming more common with the increased use of ultrasound. Data from the literature supports a high rate of spontaneous resolution and an extremely low rate of clinically relevant pathology. It is also important to note that no prospective, randomized trials are available to evaluate the various diagnostic and therapeutic options. The overall incidence of adnexal masses in pregnancy can vary greatly based on study population, use of sonography, and gestational age at presentation [1, 2]. It is reasonable to estimate that clinicians can expect to encounter adnexal masses in 2% to 10% of pregnancies [1]. Around 2% to 3% of masses removed during pregnancy are found to be malignant and this is a key issue to be considered when counseling the patient. Despite this fact, the great majority of high suspicion adnexal masses excised during pregnancy are in fact borderline ovarian tumors. Ovarian tumors of low malignant potential comprise 10–20% of all ovarian malignancies [3, 4]. They carry an excellent prognosis with 95%–99% long-term survival [3].

## 2. Case Presentation

**Figure 1:**
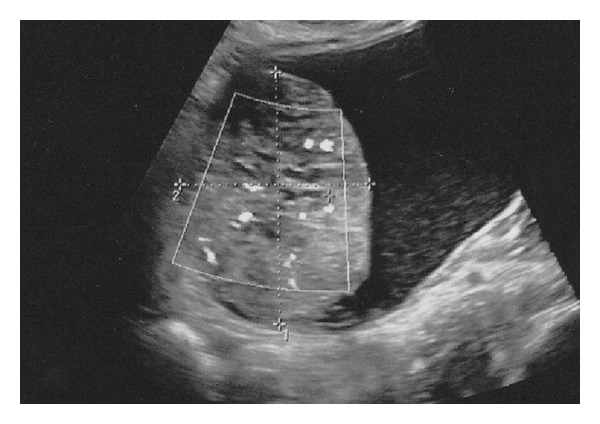
Ultrasound showing a complex mass, with a solid component.

**Figure 2:**
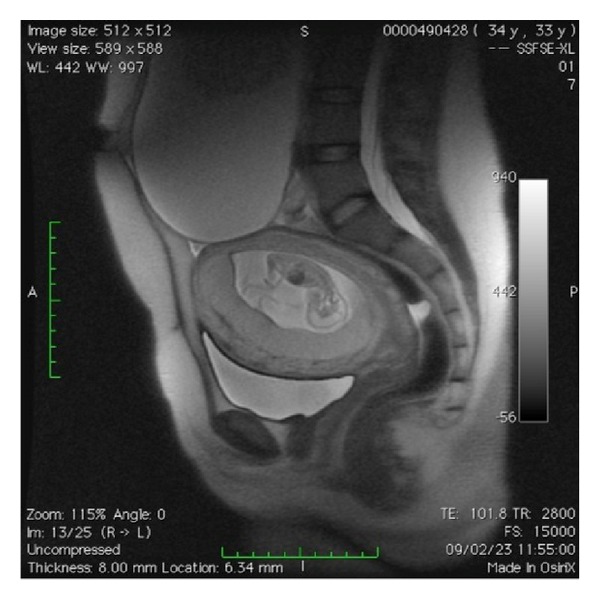
MRI demonstrating the relation between the adnexal mass and the pregnant uterus.

**Figure 3:**
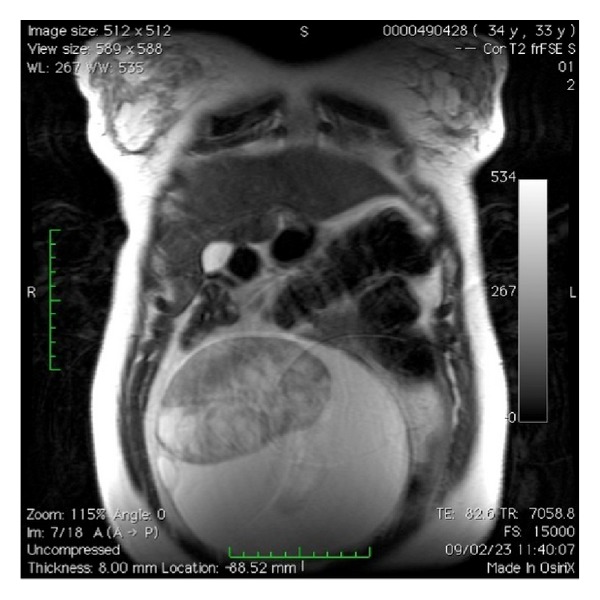
MRI showing the solid component of the adnexal mass.

We report a case of a 33-year-old gravida 2 para 1 that was referred to our department after the first trimester ultrasound scan (performed in another institution) had revealed a huge ovarian mass. The patient was asymptomatic, denying weight loss and abdominal pain or distention. A more detailed medical record was obtained and there was a positive family history of borderline ovarian tumors (BOTs). The physical examination showed a palpable abdominal mass that reached the umbilicus. The ultrasound performed in our institution showed a 12-week singleton pregnancy and an adnexal mass, measuring approximately 17 × 11.5 cm, with a heterogeneous and highly vascular solid component, arising from the right ovary (Figure 1). An abdominal magnetic resonance imaging (MRI) was performed afterwards revealing a pelvic mass, measuring 18 cm, arising from the right ovary, with a liquid component but with a major solid area (Figures 2 and 3). All laboratory data was within the normal range. CA 125 was 11.5 U/mL. Due to the high suspicion of malignancy, a laparotomy was carried out at 15 weeks of gestation (vertical midline incision). Intraoperative findings revealed a large cystic tumor of the right ovary with a solid component. Peritoneal washing was performed afterwards. The abdomen and the appendix were thoroughly examined and no signs of implants, adhesions, or metastasis were found. Right salpingo-oophorectomy was performed and the frozen section revealed a borderline mucinous ovarian tumor. Definite histology confirmed this diagnosis. The remaining pregnancy was uneventful and a cesarean section was performed at 39 weeks of gestation due to obstetrical reasons. Once again the abdomen and the appendix were systematically examined and no macroscopic changes were observed.

After discussion with the patient secondary staging surgery (peritoneal washing, total abdominal hysterectomy, unilateral salpingo-oophorectomy, omentectomy, appendectomy, and peritoneal biopsies) was performed and no evidence of disease was found. Currently the patient is being followed (36 months after the first surgery) and all imaging data show no signs of disease relapse.

## 3. Discussion

BOTs are a distinct histological entity and they account for nearly 10%–20% of all ovarian epithelial tumors [3, 4]. Approximately one-third of BOTs are diagnosed in women of childbearing age (<40 years); however little is known about the epidemiology and management of BOTs diagnosed during pregnancy. Previous studies in pregnant patients with adnexal masses have reported an incidence of BOTs ranging from 0% to 8% [2, 3].

According to the literature, BOTs are frequently diagnosed during the first trimester and usually they are detected in routine ultrasound exams. When symptomatic, patients may refer to unspecific abdominal pain [3]. 

Pelvic ultrasound remains the mainstay for evaluating the adnexa. The primary purpose is to characterize the mass and identify features that have been associated with an increased risk of malignancy, such as size >7 cm, solid components, “complex” appearance, papillary structures, internal septations, bilaterality, irregular borders, ascites, increased vascularity, and low-resistance blood flow. Bearing these features in mind, in our patient, all sonographic features were highly suspicious of malignancy. Accordingly, when there is a strong suspicion for malignancy, surgery is often indicated [1–6]. Although this approach is somewhat consensual, surgical interventions during pregnancy raise several important concerns. Women undergoing nonobstetrical surgery during pregnancy are at risk for having adverse perinatal outcomes, including preterm birth, low birth weight infants, and early neonatal death [1]. It is also important to underline that there are no established guidelines regarding the optimal timing for surgical procedures. Several groups have addressed this issue and recommendations suggest that the surgery should be performed between 14 and 22 weeks of gestation [1–3, 5, 6]. 

Another point of discussion is the surgical route. Laparotomy is usually the most definitive and traditional approach to remove adnexal masses during pregnancy. A midline vertical incision is often indicated to maximize visualization and to allow an optimal exploration of the abdomen. Bearing in mind once again the imaging features of the adnexal mass of our patient, a midline vertical laparotomy was performed not only due to the size of the tumor but also due to the high risk of malignancy. Laparoscopy may be an option because it has been shown as an effective and safe method for removing adnexal masses during pregnancy [1–3, 5, 6]. However, the size of the mass and the enlarged uterus may play an important role in the efficacy of this surgical approach. 

Confirmed low malignant potential tumors (in the frozen section examination) during pregnancy can be treated conservatively by salpingo-oophorectomy, peritoneal washing, and abdominal exploration [5]. Due to the good prognosis of these tumors, complete surgery can be deferred until after delivery [5]. However, the report by Fauvet et al. described a series of BOTs diagnosed during pregnancy with a higher incidence of aggressive features [3].

In conclusion, data from the literature is not consensual on the management of BOTs during pregnancy.
